# A curriculum focused on informed empathy improves attitudes toward persons with disabilities

**DOI:** 10.1007/s40037-013-0046-3

**Published:** 2013-03-06

**Authors:** Sonya R. Miller

**Affiliations:** 1 Physical Medicine and Rehabilitation, University of Michigan Medical School, 325 E. Eisenhower Parkway, 2nd Floor, Ann Arbor, MI 48108 USA; 2 Medical Education, University of Michigan Medical School, Ann Arbor, MI USA

**Keywords:** Advocacy, Attitudes toward persons with disabilities, Disability, Education, Empathy

## Abstract

Empathy is an important component of the provider-patient relationship. In the United States one in five persons has a disability. Persons with disabilities perceive gaps in health care providers’ understanding of their health care preferences and needs. The purpose of this study was to use valid and reliable assessment methods to investigate the association between empathy and attitudes toward persons with disabilities and advocacy. An educational module was developed to enhance health care students’ capacity for informed empathy. Pre- and post-assessment measures included the Attitude toward Disabled Persons scale (ATDP), the Attitudes toward Patient Advocacy Microsocial scale (AMIA) and the Interpersonal Reactivity Index (IRI). ATDP (*t*(94) = −5.95, *p* = .000) and AMIA (*t*(92) = −5.99, *p* = .000) scores increased significantly after the education module. Correlations between the pre- or post-module ATDP or AMIA scores and the IRI scores were not significant. Empathy in general may not be sufficient to ensure optimal attitudes toward persons with disabilities or advocacy in pre-health care professionals. However, a curriculum based on informed empathy and focused on the experiences of persons with disabilities can result in more positive attitudes toward and advocacy for people with disabilities.

## Introduction

The Center for Disease Control and Prevention estimates the prevalence of disability to be 20 % [1]. Considering the prevalence of disability in the United States increased between 2002 and 2005 and increases as individuals age [1, 2], health care providers are likely to care for patients with disabilities and therefore can benefit from an increased awareness of the needs of their patients with disabilities. The Institute of Medicine (IOM) identifies patient centredness as a core component of quality health care, and defines patient centredness as health care that establishes a partnership between practitioners, patients, and their families to ensure that decisions respect patients’ wants, needs, and preferences [3]. Patient-centred care is supported by good provider-patient communication so that patients’ needs and wants are understood and addressed [4]. However, having a disability has been found to negatively affect provider-patient communication [5–7]. Patients with disabilities report faulty communication, and express the need for better communication with health care providers [8, 9]. Individuals with disabilities want to be treated as equals in the patient-provider relationship and argue that a lack of education regarding disabilities is a major cause of miscommunication [8].

Compassion and empathy are additional components of patient-centred care. Empathy is considered a vital and important aspect of any professional helping and healing relationship, a core component of humanistic health care [10–14]. Even though there is general agreement that empathy is a critical component of any health care provider-patient relationship, it is difficult to define [15, 16]. Empathy is often considered a multidimensional construct [11, 15, 17]. Davis defined four components of empathy: (a) perspective taking (PT), the ability of the respondent to adopt the perspective or point of view of others; (b) empathic concern (EC), the tendency for the respondent to experience feelings of warmth, compassion, and concern for others undergoing a negative experience; (c) personal distress, the tendency of the respondent to experience feelings of discomfort and anxiety when witnessing the negative experiences of others; and (d) fantasy, the tendency of the respondent to identify strongly with fictitious characters in books, movies, or plays [17]. Empathy has also been defined as attunement, the process of matching emotional expressions and connectedness between two participants [18]. Larson and Yao [14] state that there should be a skill, or behavioural, dimension to empathy which reflects the interpersonal processes that happen between people, while the cognitive and affective dimensions to empathy are part of an intrapersonal process that happens within a single person.

Cox states that accurate and compassionate empathy is partly contingent on the extent to which the observer has experienced the emotions being imputed to the other [19]. Others view empathy as an attribute that enables health care providers to understand the inner experiences of patients, to communicate this understanding, and to respond in a therapeutic way [20]. Empathy facilitates the development of mutual trust, shared understanding, and optimal communication, allowing patients to feel understood and ‘listened to’ [10, 15, 16, 19]. The manner in which health care providers express empathy for persons with disabilities may contribute to their perception that their situation is not fully appreciated [21]. Ultimately, it is imperative that health care professionals learn how to adequately convey empathy because it has been linked to positive outcomes, such as reduced physiological distress, improved self-concept, reduced anxiety, and increased satisfaction with treatment [12, 18, 21]. However, because health care professionals cannot have all of the same experiences as their patients, they need other ways to gain the empathy required to provide quality care.

Most people find it easier to be empathic toward people like themselves, in part because personal experiences shape and define ones empathic understanding [14, 16]. Consequently, a training programme that captures and conveys the perspectives of specific groups, in this case persons with disabilities, may be effective in developing *informed* empathic care. For the purposes of this study, informed empathy refers to knowledge about the impairments, activity limitations, and participation restrictions that can be associated with having a disability, blended with an appreciation of the personal impact these issues can have on individuals, their families, and those who provide their care [22, 23].

Persons with disabilities report environmental and attitudinal barriers when trying to access health care [8, 24, 25]. Manifestations of attitudinal barriers are negative stereotypes, condescending or patronizing remarks, and the inability of others to see beyond the individual’s main impairment [26]. The attitudinal barriers perceived by persons with disabilities may contribute to inadequate communication from health care providers, resulting in an incomplete understanding of medical histories and a lack of thoroughness [24, 27, 28], potentially contributing to suboptimal care and health inequities for persons with disabilities.

This study involved pre-health professional students and evaluated the impact of an innovative curriculum that focused on patient-centred care for persons with disability, an area of the curriculum not historically featured in medical education. The assessment measures sought to investigate the relationship between empathy in general and attitudes toward persons with disabilities and attitudes toward advocating for patients with disabilities. It is hypothesized that: (a) a curriculum focused on informed empathy would be an effective teaching method, and (b) higher empathy scores, especially PT, would be associated with more positive attitudes toward and advocacy for people with disabilities.

## Methods

This study was approved by the medical school’s institutional review board.

### Curriculum development

The curriculum was designed to evoke reflections about attitudes, empathy, and the role of advocacy for health care professionals. To ground the educational experience in authentic representation of patients’ experience, I developed a DVD specifically for this curriculum that consisted of narratives by and about persons with disabilities. A total of 11 men and seven women with various types of disabilities were recruited from the university’s office for students with disabilities, physical medicine and rehabilitation clinics, and an association for the visually impaired and blind. I met with and obtained informed consent from each participant and explained that the DVD was being created as an educational tool. Participants were asked to share experiences or other information that they wanted current or future health care professionals to know. They were encouraged to provide an artistic interpretation, for example, a drawing, a poem, or photographs of their experiences. Everyone received a pen, notebook, disposable camera, micro-cassette recorder, and a bag to carry all of these items. Additional artistic supplies were made available when requested. Each person provided written or recorded narratives about their life and health care experiences. After reviewing their narratives, follow-up conversations were held with many of the participants to clarify their material. From the information provided, a 60-min DVD was created. It contains an oral summary of 18 narratives, each linked to one or more images. In some cases, the image is a photo, drawing, or collage that was provided by the individual. If the individual chose not to provide an image, the principal researcher and a colleague, whose formal background includes medical education and the fine arts, selected paintings from gettyimages.com. These images were selected based on the initial emotions perceived from the narratives rather than using a systematic method. Individual music compositions were recorded for each narrative and image pairing to enhance the feelings conveyed.

### Student participants

The student participants were enrolled in health-related courses and were recruited to participate in the study through web-based course sites. Informed consent was obtained online at the link to the surveys, which were administered pre- and post-module. Students who completed all pre- and post-module surveys were entered in a random draw for a $100 Visa gift card. One gift card per course was awarded. The students had approximately 2 weeks to complete the pre-module surveys and 1 week to complete the post-module surveys. Ninety-five students across seven courses completed the pre-IRI, pre- and post-ATDP scale and pre- and post-AMIA. Most of the participants were white females without a disability who were planning to enter a health profession (Table 1).

**Table 1:** Demographics of student participants

Age (years)^a^	Female, no. (%) *N* = 74 (77.9)	Male, no. (%) *N* = 21 (22.1)
18–20	23 (31.1)	0 (.0)
21–25	49 (66.2)	17 (81.0)
26–30	1 (1.4)	4 (19.0)
41 and over	1 (1.4)	0 (.0)
Race/ethnicity^b^		
Asian/Asian-American	7 (9.6)	1 (4.8)
Black/African-American	5 (6.8)	1 (4.8)
Hispanic	4 (5.5)	0 (.0)
White	54 (74.0)	18 (85.7)
Other^c^	3 (4.1)	1 (4.8)
Planning to be health care provider	*N* = 72 (78.3)	*N* = 20 (21.7)
Yes	51 (70.8)	17 (85.0)
No	14 (19.4)	2 (10.0)
Maybe	7 (9.7)	1 (5.0)
Has a disability^d^		
No	73 (98.6)	20 (95.2)
Yes^e^	1 (1.4)	1 (4.8)

^a^Age distribution differs significantly by gender (Pearson X^2^ = 17.1, df = 3, *p* = .001)

^b^No significant difference in distribution in ethnicity by gender

^c^Includes two Multiple or Other, one Lebanese, one African American/Multiracial

^d^No significant difference in disability by gender

^c^One hearing impaired, one major depressive disorder

### Curriculum implementation

The curriculum was taught at a large Midwestern university and a local community college in health-related undergraduate courses. For most of the courses, this intervention was the only curriculum content about the psychosocial aspects of disability. However, one course for dental hygienist was about patients with special needs.

The time spent teaching the curriculum ranged from 1 to 3 h. At the request of the course instructors, the principal researcher taught the curriculum in each course. The sessions began with definitions, including disability, health, patient centredness, and advocacy. It was stressed that disability is an umbrella term, making a narrow, specific definition difficult. The sessions also included discussions to engage the students about their experiences with persons with disabilities and advocacy. The students were given background information about how the DVD was made, including the fact that the narratives are in the speakers’ own words and address the following major themes: the fear and desperation they felt when their disability was diagnosed, others’ perception of disability, the desire for independence and acceptance, family support and struggles, and their experiences with medical professionals. In each session, participants spent 20–25 min viewing the DVD and then discussed its content and their impressions. The discussion was initiated by having students answer core questions such as: Which reaction/response did you understand the most or least? Which accommodations are reasonable and how much is enough?

### Measures

Assessment measures included the Attitude toward Disabled Persons (ATDP), the Attitude toward Microsocial Advocacy (AMIA), and the Interpersonal Reactivity Index (IRI). The ATDP provides an objective and reliable measure of attitudes toward persons with physical disabilities (α = .80) [29]. It was created to measure attitudes toward persons with disabilities in general, rather than toward persons with specific types of disabilities. The ATDP, developed in 1960, continues to be one of the most widely used and tested instruments to measure attitudes toward persons with disabilities [30]. The ATDP has been found to be a reliable measure across different populations, and it is sensitive to changes following instruction. It measures the attitudes of persons with and without disabilities, and validation and replication studies have identified differences in responses by gender [29]. Responses of persons without disabilities are assumed to reflect either acceptance of persons with disabilities or rejection/prejudice, depending on whether they perceive people with disabilities as similar or different and inferior. The responses of persons with disabilities are based on the assumption that most people with disabilities will respond to the questions on the ATDP by using themselves as a frame of reference, which provides information about their self-perception and perception of others with disabilities [31]. The ATDP is a self-report 20-item survey in which respondents use a six-point Likert scale, from (−3) *I disagree very much* to (+3) *I agree* *very much*, to indicate the extent of their agreement or disagreement with each item. There is no neutral point. Scores range from 0 to 120, with higher scores indicating a more favourable attitude. Individual item responses on the ATDP cannot be interpreted; only total ATDP scores are meaningful. In addition, since the ATDP uses a Likert scale, absolute interpretation of raw scores is not possible because the degree of the attitude expressed by each item is not known [31].

The Attitude toward Patient Advocacy scale was developed to evaluate nurses’ attitudes toward patient advocacy. For this scale, patient advocacy is conceptualised as a process or strategy consisting of a series of specific actions for preserving, representing, or safeguarding patients’ rights, best interests, and values. Based on this conceptual framework, patient advocacy includes safeguarding patients’ autonomy, acting on behalf of patients, and championing social justice. This scale has two subscales, the Attitude toward Macrosocial Advocacy (AMAA) and the AMIA; however, since the curriculum focuses on microsocial advocacy, only the AMIA subscale was used in the current study. The AMIA contains 45 items and responses are scored on a 6-point Likert scale ranging from (1) *strongly disagree* to (6) *strongly agree*, with a high score reflecting strong support for advocacy. In the original validity and reliability studies, the mean for the AMIA (45 items) was 244.67 (SD = 18.17) (α = .92)[32] with scores ranging from 45 to 270. For this study, the AMIA wording was modified to address patients with disabilities and two questions were combined, reducing the total number of items to 44 with scores ranging from 44 to 264.

The IRI was developed to assess the multidimensional nature of empathy. It was designed to capture individual variations in cognitive, PT tendencies as well as differences in the types of emotional reactions experienced [17]. The IRI has been found to be one of the most reliable and valid measures of self-assessed empathy [33]. It has been used with many different groups, including medical professionals. The IRI is a 28-item, self-report questionnaire consisting of four 7-item subscales, each tapping into some aspect of the global concept of empathy. IRI subscale scores range from 0 to 28, with higher scores indicating a stronger manifestation of that dimension of empathy. Respondents indicate for each question how well the item describes them. Responses are scored on a 5-point scale from (0) *does not describe me well* to (4) *describes me very well*. The four subscales are: (a) fantasy (FS), which measures the tendency of the respondent to identify strongly with fictitious characters in books, movies, or plays, for example; (b) PT, which measures the ability of the respondent to adopt the point of view of other people; (c) EC, which measures the tendency of the respondent to experience feelings of warmth, compassion, and concern for others undergoing negative experiences; and (d) personal distress (PD), which measures the tendency of the respondent to experience feelings of discomfort and anxiety when witnessing the negative experiences of others.

Significant differences between males and females on all subscales have been identified, with females having higher scores. In Davis’ normative data, the mean scores for the IRI subscales were FS = 18.75 (SD = 5.17), (α = .81); PT = 17.96 (SD = 4.85), (α = .62); EC = 21.67 (SD = 3.83), (α = .70) and PD = 12.28 (SD = 5.01), (α = .76) for females, and FS = 15.73 (SD = 5.60), (α = .79); PT = 16.78 (SD = 4.72), (α = .61); EC = 19.04 (SD = 4.21), (α = .72) and PD = 9.46 (SD = 4.55), (α = .68) for males [17]. Only scores for the individual subscales are meaningful. The IRI was not developed to provide a summation or a total score.

### Analysis

Paired *t* tests were performed to evaluate the extent of change in students’ performance on the pre- and post-module ATDP scores and AMIA scores. The IRI was only administered pre-module because the aspects of empathy measured by the IRI were not a focus of the curriculum and thus were not expected to change. Pearson correlations were performed to evaluate the magnitude of association between (a) the IRI subscales and pre- and post-ATDP scores, and (b) the IRI subscales and pre-and post-AMIA scores. This resulted in 16 different correlation tests; therefore Bonferroni’s correction for multiple tests was calculated.

## Results

Prior to instruction, there were no statistically significant differences across the courses on the students’ ATDP scores, AMIA scores, or empathy scores (Table 2). This provided empirical justification for aggregating students across courses into one group.

**Table 2:** Mean performance on attitude and empathy measures across courses

Survey	Mean of aggregated courses (95 % CI)	ANOVA across courses
Attitude toward Disabled Persons (ATDP)	79.01 (76.52–81.50)	F(6,88) = 1.72, *p* = .13
Attitude toward Patient Advocacy, Microsocial (AMIA)	225.19 (221.18–229.20)	F(6,87) = .27, *p* = .95
Empathy subscales		
Fantasy	17.55 (16.58–18.51)	F(6,88) = .94, *p* = .47
Perspective taking	19.85 (19.13–20.57)	F(6,88) = .68, *p* = .67
Empathic concern	22.08 (21.34–22.83)	F(6,88) = .38, *p* = .89
Personal distress	10.52 (9.50–11.53)	F(6,88) = 1.40, *p* = .23

Paired *t* tests showed a statistically significant increase in the ATDP and AMIA scores following the educational module (Table 3).

**Table 3:** Paired-t tests comparing the Attitudes toward Disabled Persons scale and Attitudes Toward Microsocial Advocacy scale scores

Scale	Pre-module mean	Post-module mean	*t* test equation
ATDP	79.01 (SD = 12.22)	85.56 (SD = 13.23)	*t*(94) = −5.95, *p* = .000
AMIA	225.15 (SD = 19.69)	233.70 (SD = 16.54)	*t*(92) = −5.99, *p* = .000

The mean for the pre-IRI empathy subscales were FS = 17.55 (SD = 4.73), PT = 19.85 (SD = 3.54), EC = 22.08 (SD = 3.67), and PD = 10.52 (SD = 4.96).

This study did not find significant correlations between the operationally defined dimensions of empathy and attitudes toward people with disabilities or attitudes toward advocacy. Pearson correlation between the IRI subscales and the pre-ATDP scores were: FS, r(95) = .039, *p* = .706; PT, r(95) = .182, *p* = .078; EC, r(95) = .172, *p* = .096; and PD, r(95) = −.237, *p* = .021. The correlation between the IRI subscales and the post-ATDP scores were: FS, r(95) = .039, *p* = .706; PT, r(95) = .017, *p* = .873; EC, r(95) = .164, *p* = .112; and PD, r(95) = −.116, *p* = .261. The correlation between the IRI subscales and the pre-AMIA scores were: FS, r(94) = .115, *p* = .269; PT, r(94) = .136, *p* = .192; EC, r(94) = .189, *p* = .069, and PD, r(94) = −.094, *p* = .366. The correlation between the IRI subscales and the post-AMIA scores were: FS, r(94) = .119, *p* = .254; PT, r(94) = .017, *p* = .873; EC, r(94) = .123, *p* = .238; and PD, r(94) = −.045, *p* = .670. The largest magnitude of correlation emerged between personal distress and the pre-ATDP score, but it failed to meet the alpha level correction for multiple tests, Bonferroni’s correction (.05/16 = .003).

## Discussion

This study established the feasibility of education involving authentic representation of persons with disabilities and student self-reflection. The active engagement of students encouraged them to self-reflect and consider the challenges people with disabilities face in general and when obtaining health care. This innovative educational module involved patients in the curriculum design and curricular material, an example of authentic patient-centred education. The curriculum resulted in a significant increase in ATDP and AMIA scores, well-established assessment measures, possibly through the process of gaining informed empathy.

The DVD was a powerful contributor to the effectiveness of the curriculum. This likely reflects the students’ recognition that the DVD authentically portrayed the experiences of persons with disabilities. Further, the DVD included a diversity of characteristics, which contributed to the likelihood that students were able to identify with some aspect of the narratives, an initial step in developing informed empathy for persons with disabilities. For example, it may have been the age, ethnicity, or type of disability of a DVD participant; it may have been a reference to an area, or restaurant that the student goes to or is familiar with; it may have been an experience or activity the DVD participant was unable to access or do that the student does regularly without difficulty, such as using public transportation.

The results, however, did not support the hypothesis that higher empathy scores, specifically PT, would correlate with higher ATDP or AMIA scores. Compared with the means reported by Davis, the students’ scores on the empathy subscales were equivalent to the normative data [17]. The scores for the IRI empathy subscales were not correlated with the pre- or post-module ATDP or AMIA scores. This may be because the IRI is not sensitive to the issues included on the ATDP or AMIA. Measuring different dimensions of empathy or a global measure of empathy may be better associated with attitudes toward and advocacy for persons with disabilities.

There was already good evidence of the reliability of the ATDP, AMIA, and IRI; however, I sought to explore their associations with a very relevant conceptual framework developed outside the domain of medicine. The narratives in the DVD are not limited to medical scenarios; of the five major themes identified in the DVD narratives through qualitative analysis, only one was related to experiences with medical professionals. Consequently, class discussions were not limited to the interactions that a person with a disability may have with medical personnel or a health system. The students were encouraged to consider and discuss interactions (experienced or observed) with individuals with disabilities and the attitudes expressed, reactions witnessed, and barriers and opportunities identified. This is important since health is influenced by more than a diagnosis, disability, medical professional, or hospital. In an effort to capture these possible aspects of influence, most of the assessment tools are not specific to medicine (e.g., ATDP and IRI). And although the AMIA is specific to health care, the classroom discussions about advocacy extended beyond medicine. This created an opportune setting to teach about advocacy, which has been identified as a component of professionalism [34, 35] and is receiving increased attention in medical education. A patient-centred approach toward advocacy education allowed the students to discern examples of advocacy that may be especially pertinent to individuals with disabilities.

When creating the DVD there was a focus on eliciting participants’ experiences about the health care they received, and on any life experiences they felt were important for current or future health care professionals to know. Participants were encouraged to tell their stories in their own words. This allowed them to emphasize the actions, attitudes, and feelings that were important to them. By explaining the methods and reasoning used to create the DVD, I modelled for the students a method of helping persons with disabilities feel ‘listened to’. This innovative educational module allowed the speakers to be regarded as individuals with unique concerns, not merely a disability or illness to be ‘fixed’.

The curriculum utilizes stories, art, paintings, images, guided discussions about shared experiences and feelings, and self-reflection to help students understand people who may be very different from themselves. Course instructors have observed that this unique curriculum ‘got students to open their minds when considering the barriers to caring for their patients.’ Students have commented that ‘class participation is both enriching and thought provoking.’ This study demonstrates, as other studies have, that literature, film, and art are effective in developing and enhancing informed empathy [13, 14].

Future studies could include transcribing class discussions and using qualitative analysis to better understand the process that is contributing to the improved attitude scores. Another consideration is the possible longitudinal effects of the curriculum, especially on clinical practice. Providers, who as students were trained using this curriculum, may develop better communication with individuals with disabilities, resulting in a more therapeutic relationship and improved satisfaction of care for both the patient and provider. Using different tools to assess attitudes and empathy may provide additional information on the effectiveness of the curriculum. In addition, since the expression of empathy by providers and the empathic needs of patients can vary based on the situation, gender, ethnicity, or age, exploring how these areas intersect and influence attitudes could help health care providers to better understand their own reactions, responses, and biases. Studies involving a more balanced number of males and females may determine the effect, if any, that gender has on pre- and post-assessment scores. Lastly, using direct observation to assess attitudes toward real patients with disabilities could provide information about the effectiveness of the curriculum on improving/maximizing attitudes and communication in the clinical setting.

### Strengths and limitations of study

Strengths of this study are (a) participants were students from a variety of pre-health courses, (b) the use of well-established assessment measures, and (c) matched pre- and post-education comparisons. This study is limited by the relatively small number of participants, its cross-sectional methodology, and the use of questionnaires, which may have resulted in socially desirable answers. Additionally, students were recruited from only two sites. Most participants were white females without disabilities; therefore, the results may not be generalized to other populations. This study did not assess the long-term influence of the educational module.

## Essentials

- Patient-centred education is an effective teaching method.
- Persons with disabilities are effective, compelling narrators.
- Attitudes toward and advocacy for individuals with disabilities can be enhanced through informed empathy.
- Informed empathy can be tailored toward specific groups.

## References

[1] Center for Disease Control and Prevention. Prevalence of disability among adults, United States 2008; 2008. http://www.cdc.gov/ncbddd/disabilityandhealth/data.html. Accessed 18 Jun 2012.

[2] Brault M (2008). Americans with disabilities: 2005, current population reports.

[3] Institute of Medicine (2001). Crossing the quality chasm: a new health system for the 21st century.

[4] Institute of Medicine (2001). Envisioning the national health care quality report.

[5] Smith DL (2009). Disparities in patient-physician communication for persons with a disability from the 2006 Medical Expenditure Panel Survey (MEPS). Disabil Health J.

[6] Agency for Healthcare and Research Quality (2010) National healthcare disparities report, 2010; 2008. www.ahrq.gov/qual/nhdr10/nhdr10.pdf. Accessed 22 July 2011.

[7] Drainoni M, Lee-Hood E, Tobias C, Bachman S, Andrew J, Maisels L (2006). Cross-disability experiences of barriers to health-care access. J Disabil Policy Stud.

[8] Morrison EH, George V, Mosqueda L (2008). Primary care for adults with physical disabilities: perception from Consumer and Provider Focus Groups. Fam Med.

[9] Becker H, Stuifbergen A, Tinkle M (1997). Reproductive health care experiences of women with physical disabilities: a qualitative study. Arch Phys Med Rehabil.

[10] Suchman AL, Matthews DA (1988). What makes the patient-doctor relationship therapeutic? Exploring the connexional dimension of medical care. Ann Int Med.

[11] Reynolds WJ, Scott B, Jessiman WC (1999). Empathy has not been measured in clients’ terms or effectively taught: a review of the literature. J Adv Nurs.

[12] Rogers CR (1975). Empathic: an unappreciated way of being. Counsel Psychol.

[13] Shipro H (1992). What is empathy and can it be taught?. Ann Int Med.

[14] Larson EB, Yao X (2005). Clinical empathy as emotional labor in the patient-physician relationship. J Am Med Assoc.

[15] Reynolds S, Austin W (2000). Nursing, empathy and perception of the moral. J Adv Nurs.

[16] Williams J, Stickley T (2010). Empathy and nurse education. Nurs Educ Today.

[17] Davis MH (1980). A multidimensional approach to individual differences in empathy. Catalog Sel Documents Psychol.

[18] Moore LA (2010). Being empathetic: benefits and challenges for the clinician and client. Top Stroke Rehabil.

[19] Cox JL (2011). Empathy, identity and engagement in person-centred medicine: the sociocultural context. J Eval Clin Pract.

[20] Smajdor A, Stöckl A, Salter C (2011). The limits of empathy: problems in medical education and practice. J Med Ethics.

[21] Reynolds WJ, Scott B (2000). Do nurses and other professional helpers normally display much empathy?. J Adv Nurs.

[22] Young A, Connor-Greene P, Waldvogel J, Paul C (2003). Poetry across the curriculum: four disciplinary perspectives. Lang Learn Across Discipl.

[23] Connor-Greene PA, Murdoch JW, Young A, Paul C (2005). Poetry: it’s not just for english class anymore. Teach Psychol.

[24] Smeltzer SC, Sharts-Hopko NC, Ott BB, Zimmerman V, Duffin J (2007). Perspectives of women with disabilities on reaching those who are hard to reach. J Neurosci Nurs.

[25] Nosek M, Hughes R, Howland C, Young M, Mullen P, Shelton M (2004). The meaning of health for women with physical disabilities: a qualitative analysis. Fam Community Health.

[26] Thomas C (2001). Medicine, gender, and disability: disabled women’s health care encounters. Health Care Women Int.

[27] Iezzoni LI (2006). Make no assumptions: communication between persons with disabilities and clinicians. Assist Technol.

[28] Iezzoni LI, Davis RB, Soukup J, O’Day B (2003). Quality dimensions that most concerned people with physical and sensory disabilities. Arch Intern Med.

[29] Yuker HE, Block JR, Youung J (1970). The measurements of attitudes towards disabled persons. Human resources center.

[30] Lam W, Gunukula S, McGuigan D, Isaiah N, Symons A, Akl E (2010). Validated instruments used to measure attitudes of healthcare students and professionals towards patients with physical disability: a systematic review. J NeuroEng Rehabil.

[31] Yuker HE, Block JR (1986). Research with the attitudes towards disabled persons scales (ATDP) 1960–1985.

[32] Bu X, Wu YB (2008). Development and psychometric evaluation of the instrument: attitude toward patient advocacy. Res Nurs Health.

[33] Neumann M, Edelhäuser F, Tauschel D (2011). Empathy Decline and Its Reasons: A Systematic Review of Studies With Medical Students and Residents. Academic Medicine.

[34] American Medical Association. Declaration of professional responsibility: medicine’s social contract with humanity. 2001; http://www.ama-assn.org/ama/upload/mm/369/decofprofessional.pdf. Accessed November 23, 2011. 12025762

[35] ABIM Foundation, ACP-ASIM Foundation, European Federation of Internal Medicine (2002). Medical professionalism in the new millennium: a physician charter. Ann Int Med.

